# Comparison of Three Radiobiological Models in Stereotactic Body Radiotherapy for Non-Small Cell Lung Cancer

**DOI:** 10.7150/jca.33001

**Published:** 2019-08-08

**Authors:** Jia-Yang Lu, Zhu Lin, Pei-Xian Lin, Bao-Tian Huang

**Affiliations:** 1 Department of Radiation Oncology, Cancer Hospital of Shantou University Medical College, Shantou 515031, Guangdong, China; 2 Department of Nosocomial Infection Management, The Second Affiliated Hospital of Shantou University Medical College, Shantou 515041, Guangdong, China

**Keywords:** radiobiological models, stereotactic body radiotherapy, non-small cell lung cancer

## Abstract

**Objective:** The applicability of the linear quadratic (LQ) model to local control (LC) modeling after hypofractionated radiotherapy to treat lung cancer is highly debated. To date, the differences in predicted outcomes between the LQ model and other radiobiological models, which are characterized by additional dose modification beyond a certain transitional dose (d_T_), have not been well established. This study aims to compare the outcomes predicted by the LQ model with those predicted by two other radiobiological models in stereotactic body radiotherapy (SBRT) for non-small cell lung cancer (NSCLC).

**Methods:** Computer tomography (CT) simulation data sets for 20 patients diagnosed with stage Ⅰ primary NSCLC were included in this study. Three radiobiological models, including the LQ, the universal survival curve (USC) and the modified linear quadratic and linear (mLQL) model were employed to predict the tumor control probability (TCP) data. First, the d_T_ values for the USC and mLQL models were determined. Then, the biologically effective dose (BED) and the predicted TCP values from the LQ model were compared with those calculated from the USC and mLQL models.

**Results:** The d_T_ values from the USC model were 29.6 Gy, 33.8 Gy and 44.5 Gy, whereas the values were 90.2 Gy, 84.0 Gy and 57.3 Gy for the mLQL model for 1-year, 2-year and 3-year TCP prediction. The remarkable higher d_T_ values obtained from the mLQL model revealed the same dose-response relationship as the LQ model in the low- and high-dose ranges. We also found that TCP prediction from the LQ and USC models differed by less than 3%, although the BED values for the two models were significantly different.

**Conclusion:** Radiobiological analysis reveals small differences between the models and suggested that the LQ model is applicable for modeling LC using SBRT to treat lung cancer, even when an extremely high fractional dose is used.

## Introduction

The efficacy of stereotactic body radiotherapy (SBRT) for early-stage non-small cell lung cancer (NSCLC) has been fully validated 1-5. It has been reported that SBRT achieves promising outcomes comparable to those of surgery 6-8.

Although using SBRT to treat early-stage lung cancer has been a great success, the applicability of the linear quadratic (LQ) model for modeling local control (LC) after hypofractionated radiotherapy is currently controversial 9, 10. Recently, several independent studies have reported the applicability and reliability of traditional LQ formalism for modeling the dose-response relationship of SBRT for stage Ⅰ NSCLC 11-13. In contrast, Park et al. found that the universal survival curve (USC), which uses the LQ model in the low-dose range and transits to a linear dose-response relationship beyond a certain transitional dose (d_T_), better simulated the experimentally measured survival curves than the traditional LQ model 14. To our knowledge, the differences between the outcomes predicted using the traditional LQ model and other radiobiological models that require additional dose modification beyond certain d_T_ values have not been elucidated.

This study aims to compare the predicted data from three radiobiological models and to find whether the LQ model is applicable for modeling LC for NSCLC undergoing SBRT.

## Materials and Methods

### Ethics statement

The work of computer tomography (CT) image acquisition was approved by the Clinical Research Ethics Review Committee of Cancer Hospital of Shantou University Medical College. Since this was a retrospective dosimetric study involving no impact on individuals, the Ethics Review Committee had abandoned the requirement for written approval of this study.

### Immobilization and CT scanning

A total of 20 patients diagnosed with peripheral stage Ⅰ NSCLC were included in the study. The patients were immobilized in the supine position with a vacuum bag (Medtec Medical, Inc, Buffalo Grove, IL) or a thermoplastic mask (Guangzhou Klarity Medical & Equipment Co., Ltd, Guangzhou, People's Republic of China). All of the patients were simulated using a Brilliance Big Bore CT (Philips Brilliance CT Big Bore Oncology Configuration, Cleveland, OH, USA) under free breathing conditions. CT scan was performed at a 3-mm slice thickness during scanning using respiratory-correlated four-dimensional computed tomography (4DCT) via a Real-time Position Management System (Varian Medical System, Inc., Palo Alto, CA). The CT images, including the reconstructed maximum intensity projection (MIP) and average intensity projection (AIP) images, were transferred to Eclipse treatment planning system (Version 10.0, Varian Medical System, Inc., Palo Alto, CA) for target and organs at risk (OARs) delineation, treatment planning and treatment plan evaluation.

### Target delineation and OARs contouring

The internal target volume (ITV) was contoured to cover all the gross tumor volume (GTV) on ten phases of the 4DCT scans under the pulmonary windows. A planning target volume (PTV) was created by adding 5-mm margin around the ITV to account for set-up uncertainties and potential baseline tumor shifts. Regarding to the OARs contouring, the whole lung was limited to the air-inflated lung parenchyma, and the GTV and trachea/ipsilateral bronchus were excluded according to the Radiation Therapy Oncology Group (RTOG) 0915 report 15. The chest wall (CW) was segmented from the corrected lung edges with 2 cm expansion in the lateral, anterior, and posterior directions, excluding the lung volume and the mediastinal soft tissue. The 2-cm expanded structure was also restricted within the body. To avoid complicated delineation of the entire CW, we defined it within a 3-cm limit in the head-to-feet direction from the PTV 16.

### Treatment planning

A 1×30 Gy fraction regime was prescribed in the study; 1×30 Gy represents 30 Gy treated in 1 fraction. We inferred that if the LQ model was proved to applicably model the dose-response relationship using this fraction regime, it was undoubtedly appropriate to model SBRT for stage Ⅰ NSCLC with other fraction regimes. The reason was commented in the discussion section in detail. The treatment planning was performed on AIP images in the Eclipse treatment planning system. All of the plans were designed using TrueBeam linear acceleration with a 6-MV flattening filter free (FFF) photon beam and a maximum dose rate of 1400 MU/min. Dual partial arcs were established to protect the contralateral lung from irradiation. The collimator angles for all of the plans were set to 30° and 330°, aiming to minimize the contribution of the tongue-and-groove effect to the dose. The progressive resolution optimizer (PRO, version 10.0.28) algorithm was applied for optimization. The objectives were adjusted to ensure that the maximum dose was centered on the GTV. The final dose was calculated using the anisotropic analytical algorithm (AAA, version 10.0.28) with a grid resolution of 1 mm while accounting for heterogeneity correction. All of the plans were optimized to attain clinically acceptable PTV coverage and OAR sparing as mentioned in RTOG 0915 protocol and other publications 15, 17. Because different dose prescriptions result in different maximum dose (D_max_) at the target and ultimately influence tumor control probability (TCP) , four gradients of dose prescriptions were generated as follows: (1) the D_max_ was approximately 120% of the prescribed dose (referred to as P_120%_), (2) the D_max_ was approximately 115% of the prescribed dose (referred to as P_115%_), (3) the D_max_ was approximately 110% of the prescribed dose (referred to as P_110%_), (4) the D_max_ was approximately 105% of the prescribed dose (referred to as P_105%_).

### Determination of the d_T_ values from the USC and modified linear quadratic and linear (mLQL) models

Both the USC and mLQL models include a transitional dose threshold to model the dose-response relationship at high fractional doses and we needed to determine the d_T_ threshold before calculating the TCP values. The parameters used to determine d_T_ values for 1-year, 2-year and 3-year TCP prediction in the USC model were obtained from Liu's research. In detail, α, D_0_ (-1/D_0_ is the slope of the logarithmic survival curve) and D_q_ (the x-intercept of the logarithmic survival curve) for 1-year TCP prediction were 0.215, 1.1 and 11.3, respectively. α, D_0_ and D_q_ for 2-year TCP prediction were 0.185, 1.5 and 12.2, respectively. The corresponding values for 3-year TCP prediction were 0.163, 1.7 and 16.1. The d_T_ values was calculated as follows: d_T_=2D_q_/(1-αD_0_). The d_T_ values for 1-year, 2-year and 3-year TCP calculations in the mLQL model were also based on Liu's work 18.

### Comparison of biologically effective dose (BED) and TCP between the LQ and USC models with a high fractional dose schedule

The 1-year, 2-year and 3-year BED and TCP data predicted using the LQ and USC models were calculated using the isocentric dose rather than the PTV encompassing dose as a predictor 11. The physical dose of the tumor was first converted to the BED. For the LQ model, the BED^LQ^=D×(1+d/α/β), in which α/β characterizes the intrinsic radiosensitivity of cells, and D and d are the total and fractional doses, respectively. The α/β values for 1-year, 2-year and 3-year BED conversion were 17.9 Gy, 26.0 Gy and 32.5 Gy, respectively. For the USC model, BED^USC^=(D-n×D_q_)/(α×D_0_), when d≥d_T_. The α, D_0_ and D_q_ values for 1-year, 2-year and 3-year BED and TCP calculations were the same as those for the d_T_ calculations in the previous section. For both models, the TCP values were calculated with the same formula, TCP=e^-K0×e(-α×BED)^. The K_0_ values were obtained via private communication with the author. Calculations of the BED and TCP values were performed using an in-house program developed with MATLAB 2012 (MathWorks, USA).

### Statistical analysis

The BED values are presented as the means±standard deviations (SD). The TCP values are expressed as the medians (ranges). The differences between BED between for the LQ and USC models were assessed with a two-tailed Student's t-test, and the TCP values were compared using a matched-pair Wilcoxon signed-rank test in SPSS 19.0 (Chicago, IL). The data were considered statistically significant when the *p*-value was <0.05.

## Results

### Patient characteristics

Twelve T1 (60%) and eight T2 (40%) staged peripheral NSCLC patients were recruited for this study. Twelve of the patients were men, and the remaining patients were women. The average age was 65.6±7.3 years old. The average tumor diameter and tumor volume were 2.5±0.8 cm and was 12.5±16.2 cc, respectively. Patient characteristics were described in Table 1.

### d_T_ values from the USC and mLQL models

Table 2 summarized the d_T_ values for predicting 1-year, 2-year and 3-year TCP statistics with the USC and mLQL models. The d_T_ values from the mLQL model were significantly higher than those from the USC model. The d_T_ values from the USC model for 1-year, 2-year and 3-year TCP prediction were 29.6 Gy, 33.8 Gy and 44.5 Gy, respectively. The d_T_ values from the mLQL model for 1-year, 2-year and 3-year TCP prediction were 90.2 Gy, 84.0 Gy and 57.3 Gy, respectively.

### BED differences between the LQ and USC models

**Figure 1:**
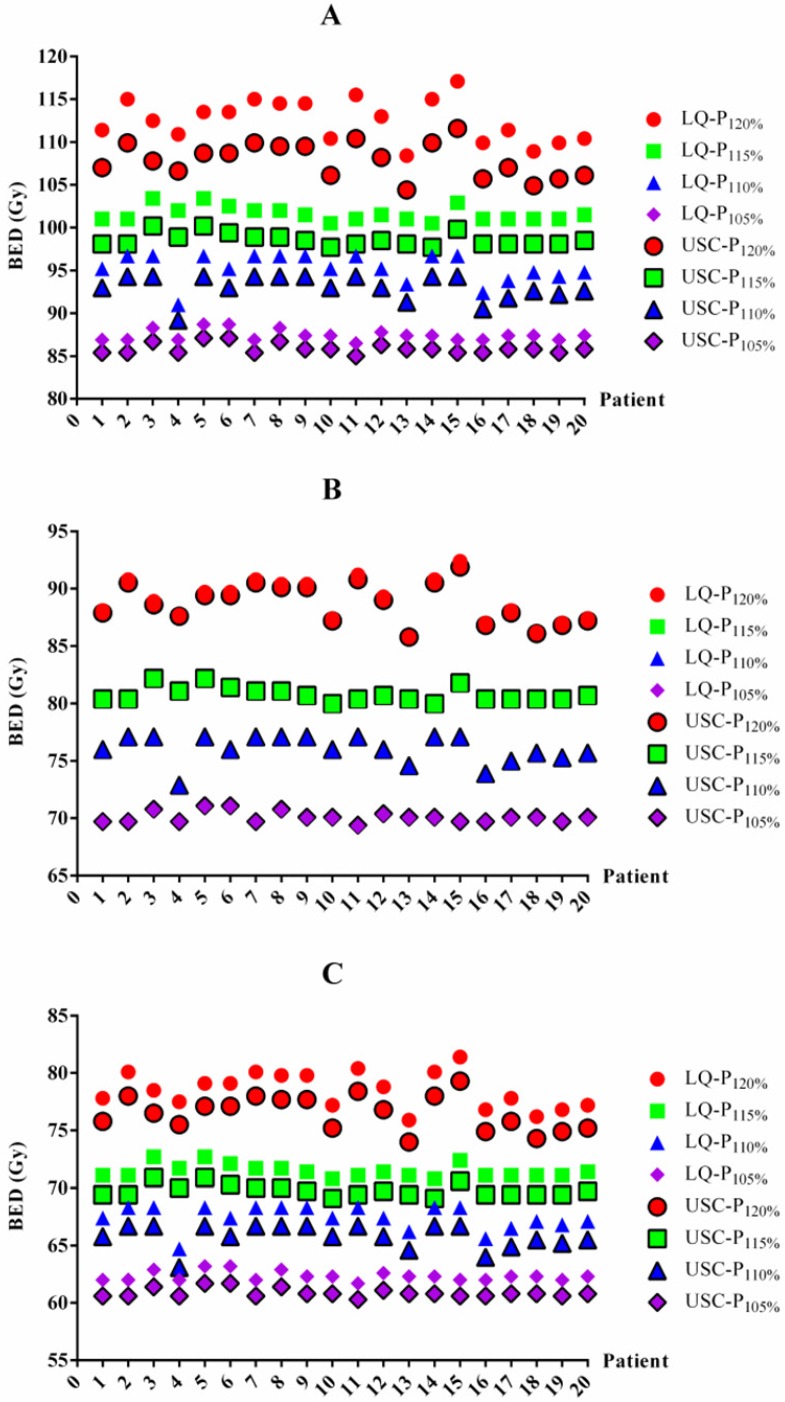
BED values for each patient based on the LQ and USC models. BED values for 1-year (A), 2-year (B) and 3-year (C) TCP prediction. BED=biologically effective dose. LQ=linear quadratic model. USC=universal survival curve model. P_120%_, P_115%_, P_110%_ and P_105%_= maximum dose of 120%, 115%, 110% and 105% of the prescribed dose, respectively.

The BED differences between the LQ and USC models were listed in Table 3. For 1-year TCP prediction, the BED values from the LQ model in groups P_120%_, P_115%_, P_110%_ and P_105%_ were 4.6 Gy, 3.0 Gy, 2.3 Gy and 1.5 Gy higher on average, respectively, than those of the USC model. For 2-year TCP prediction, the BED value from the LQ model for group P_120%_ was 0.3 Gy higher on average than that from the USC model. However, the results for groups P_115%_, P_110%_ and P_105%_ were exhibited 0.3 Gy lower on average than those calculated from the USC model. Similar to the 1-year data, the BED values from the LQ model for groups P_120%_, P_115%_, P_110%_ and P_105%_ were 2.0 Gy, 1.8 Gy, 1.6 Gy and 1.5 Gy higher on average, respectively, than those from the USC model for 3-year TCP prediction. All of the comparisons were statistically significant, although some of the differences were small. The BED values for each patient from the LQ and USC models were displayed in Figure 1.

### TCP differences between the LQ and USC models

**Figure 2:**
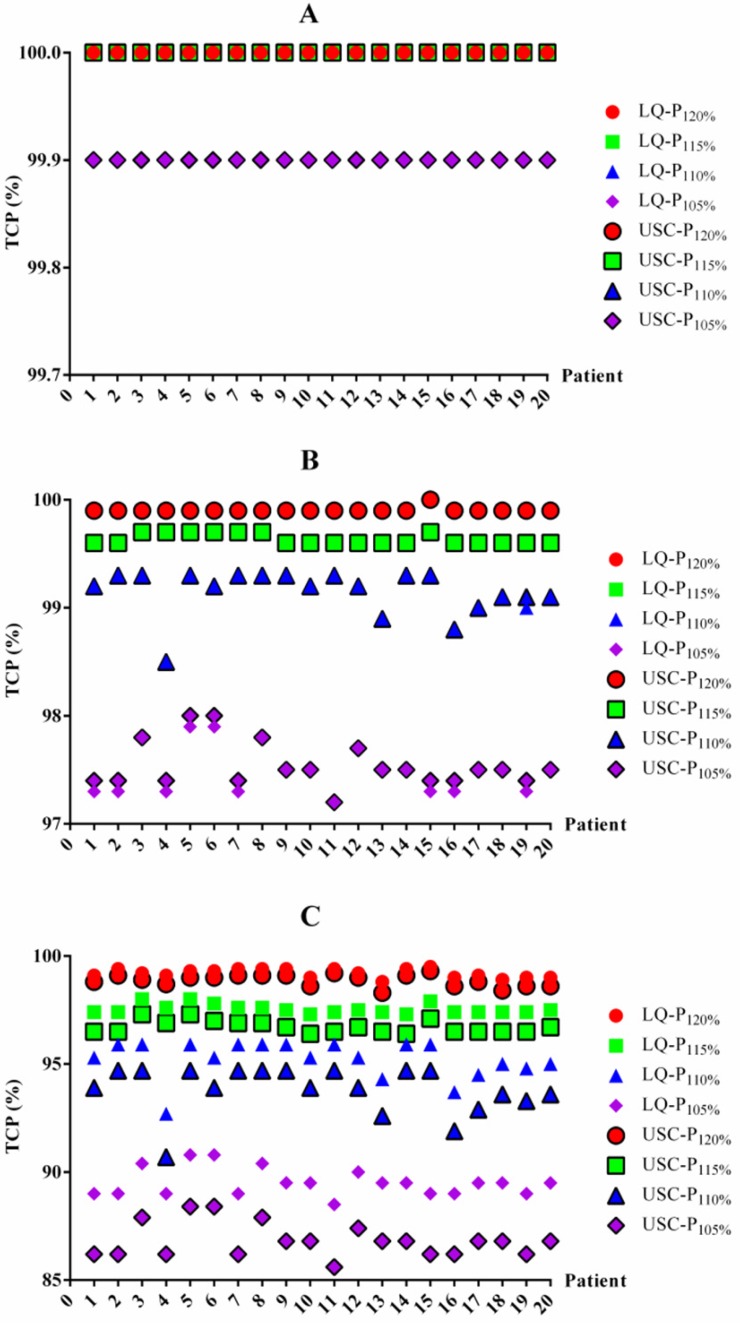
The TCP values for each patient based on the LQ and USC models. TCP values for 1-year (A), 2-year (B) and 3-year (C) TCP prediction. TCP=tumor control probability. LQ=linear quadratic model. USC=universal survival curve model. P_120%_, P_115%_, P_110%_ and P_105%_= maximum dose of 120%, 115%, 110% and 105% of the prescribed dose, respectively.

To determine whether the BED differences translated into TCP changes, we also summarized the TCP differences between the LQ and USC models (Table 4). For 1-year and 2-year TCP prediction, the data from the LQ model for groups P_120%_, P_115%_, P_110%_ and P_105%_ were almost the same as those from the USC model, although the data from group P_105%_ exhibited a slight difference. For 3-year TCP prediction, the median TCP values from the LQ model for groups P_120%_, P_115%_, P_110%_ and P_105%_ were only 0.2%, 0.9%, 1.4% and 2.7% higher, respectively, than those calculated using the USC model. All of the comparisons for 3-year prediction were statistically significant. The TCP value for each patient from the LQ and USC models were displayed in Figure 2.

## Discussion

The predicted outcomes between the LQ model and other radiobiological models that require additional dose modification beyond a certain d_T_ have not been well established. To address this question, we first determined d_T_ values using the USC and mLQL models and then compared the resultant LC data with that from the traditional LQ model using radiobiological analysis.

Our analysis demonstrated small differences in TCP prediction between different radiobiological models, suggesting that the LQ model is applicable for modeling the LC in SBRT for lung cancer even at extremely high fractional doses. To the best of our knowledge, this is the first study to compare the data predicted using the LQ, USC and mLQL models in SBRT for NSCLC using radiobiological analysis.

A variety of single and multiple fractions SBRT dose schedules were widely used to treat lung cancer in the clinic, including 30 Gy in a single fraction, 34 Gy in a single fraction, 45 Gy in 3 fractions, 54 Gy in 3 fractions, 60 Gy in 3 fractions, 48 Gy in 4 fractions, and so on. Because the minimum d_T_ value from the USC and mLQL models was 29.6 Gy (Table 2), the LQ model without additional dose modification beyond a specific dose threshold was no doubt suitable to model hypofractionated radiation therapy for lung cancer at most dose schedules, except for 30 Gy and 34 Gy in a single fraction. Although 34 Gy in single fraction is the highest fractional dose currently used to treat lung SBRT, a subsequent study demonstrated that a dose schedule of 30 Gy in single fraction achieves similar outcomes as 34 Gy in single fraction with respect to overall efficacy, failure, cause of death, and overall survival 19. Additionally, we observed that the d_T_ values for 1-year, 2-year and 3-year TCP predictions in the USC model were 29.6 Gy (98.7% of the prescription), 33.8 Gy (112.7% of the prescription) and 44.5 Gy (148.3% of the prescription), respectively, and these values are potentially obtainable during clinical treatment when using the 30 Gy in single fraction dose schedule. For this reason, only the dose schedule of 30 Gy in single fraction was employed in this analysis. Because the d_T_ values from the mLQL model were far beyond the clinical dose distribution for lung SBRT, only comparisons for the BED and TCP values between the USC and the traditional LQ models were performed in this study. In general, the TCP prediction in the study was the largest difference among the LQ, USC and mLQL models. The same BED and TCP prediction will be found in the three models when other fractionated dose regimes were applied.

If the TCP values calculated from the USC model are similar to those using the traditional LQ model, it fully demonstrates that the LQ model is applicable to all investigated dose schedules. Consistent with the general recognition that the LQ model might overestimate the effect of high fractional radiation doses, our analysis demonstrated that the BED values from the LQ model were 1.5-3.6 Gy higher than those from the USC model for 1-year or 3-year estimations; however, the BED differences did not translate into changes in TCP, which differed less than 3% between the two models. Ruggieri et al. reported that an isocentric fractional dose of up to 20 Gy was best predicted using the TCP model based on LQ after analyzing the clinical outcomes of early-stage NSCLC treated with SBRT 20. Importantly, our results prove that the LQ model is applicable even at high fractional doses of up to 30 Gy. The applicability of LQ model was extremely essential for clinical application when performing the BED conversion or optimal fractionation scheme determination.

Inhomogeneous dose distributions frequently occur during lung SBRT and involve maximum isocentric doses of 105% to >150% of the prescribed PTV dose 11. The inhomogeneous dose distribution causes the isocentric doses to vary from study to study and ultimately influences the outcome, as several investigations have reported that the maximum isocentric dose to the tumor is highly correlated with LC of the tumor 11, 12, 18. Therefore, four gradients of dose prescriptions were generated in this study to distinguish their influence. Notably, we observed that the differences between the BED and TCP values from the LQ and USC models revealed consistent changes across the four gradients of dose prescriptions, indicating that the isocentric dose had little influence on our results.

Park et al. found that the USC model best described the survival curve of the H460 cell line in the ablative dose range beyond the shoulder. These authors also reported that the mean d_T_ value was 6.2 Gy in 12 NSCLC lines 14. However, the 1-year, 2-year and 3-year d_T_ values from the USC model calculated in our study deviated widely from Park's results. Since the parameters used in our study were generated from clinical data of 3479 patients but the d_T_ value of 6.2 Gy from Park et al. was based only on fitting the results of an *in vitro* cell experiment, the d_T_ value determined in our study was more convincing.

The applicability of the LQ model to radiobiological modeling has been questioned recently; however, the models' accuracy has been confirmed by numerous clinical studies. Guckenberger et al. found that the traditional LQ formalism accurately modeled the LC data for 395 patients with stage Ⅰ NSCLC received SBRT from 13 German and Austrian centers 11. Shuryak et al. reported that the LQ model provided a significantly better fit to the LC data from NSCLC patients than did any of the models that require additional terms at high-dose ranges 13. Santiago et al. demonstrated that the LQ model was adequate to simulate LC after hypofractionated irradiation and was a robust method for predicting 3-year LC data by analyzing 1975 patients 12. Fowler et al. suggested that the LQ model with large α/β ratio was applicable to model the local TCP of early stage lung cancer treated with SBRT 21. Similarly, Liu et al. explored a series of radiobiological models by fitting the published clinical TCP data of early stage NSCLC and found large α/β ratio of 20 Gy could well model the TCP values 18. The accuracy of the conventional LQ model has gradually become recognized in accumulating studies. Consistent with published papers, our study also demonstrates that the traditional LQ model is feasible for modeling LC after SBRT treatment for lung cancer even at very high fractional doses.

Although our study have demonstrated that the traditional LQ model is applicable for evaluating SBRT outcome for lung cancer even at high fractional doses, there are some limitations. (1) The highest dose prescription was only 120% of the prescribed dose in our study, and this may partially weaken our results because the maximum dose reported was >150% of the prescribed dose for lung SBRT. Whether the traditional LQ model is also applicable at very high-dose prescriptions is another topic for future work. (2) Recently, several studies have validated the traditional LQ model with clinical data; however, the results were based on a mixture of dose schedules and few retrospective studies of single-fraction SBRT dose schedules were reported. Therefore, the d_T_ values in single dose schedules can not be determined and it is difficult to compare our results with clinical outcomes. In other words, large clinical samples of patients treated with single-fraction SBRT are required to further validate our results because the effect of hypoxia in single fraction TCP data for lung SBRT is still unclear 22.

In summary, the study find small differences between radiobiological models by comparing the TCP predictions calculated from the LQ, USC and mLQL models. Further validation of SBRT treatment for lung cancer in clinical practice is necessary.

## Figures and Tables

**Table 1:** Basic information of the 20 NSCLC patients receiving SBRT

Patient	Gender	Age	Stage^*^
1	F	71	T2
2	M	71	T1
3	M	68	T1
4	F	72	T1
5	M	64	T1
6	M	70	T1
7	M	62	T2
8	F	63	T1
9	F	70	T1
10	M	68	T1
11	M	62	T1
12	F	59	T1
13	M	61	T1
14	F	76	T1
15	M	72	T2
16	F	56	T2
17	M	51	T2
18	F	66	T2
19	M	51	T2
20	M	72	T2

^*^ According to American Joint Committee on Cancer (AJCC), 7^th^ edition. M=Male; F=Female.

**Table 2:** d_T_ values from the USC and mLQL models

TCP	USC	mLQL
1-year	29.6 Gy	90.2 Gy
2-year	33.8 Gy	84.0 Gy
3-year	44.5 Gy	57.3 Gy

TCP=tumor control probability. USC=universal survival curve model. mLQL=modified linear quadratic and linear model.

**Table 3:** BED values in the LQ and USC models

BED	LQ					USC			
	P_120%_	P_115%_	P_110%_	P_105%_		P_120%_	P_115%_	P_110%_	P_105%_
1-year	112.5±2.5^a^	101.6±0.9^a^	95.3±1.6^a^	87.4±0.6^a^		107.9±2.0	98.6±0.8	93.0±1.5	85.9±0.6
2-year	89.0±1.9^b^	80.7±0.7^b^	76.0±1.2^b^	70.0±0.5^b^		88.7±1.7	80.8±0.7	76.1±1.2	70.1±0.5
3-year	78.5±1.6^c^	71.5±0.6^c^	67.4±1.1^c^	62.4±0.4^c^		76.5±1.5	69.7±0.6	65.8±1.0	60.9±0.4

Data were presented as mean ± SD. ^a^ statistically significant with *p* value < 0.05 compared with the USC model for 1-year BED calculation. ^b^ statistically significant with *p* value < 0.05 compared with the USC model for 2-year BED calculation. ^c^ statistically significant with *p* value < 0.05 compared with the USC model for 3-year BED calculation. BED=biologically effective dose. LQ=linear quadratic model. USC=universal survival curve model. P_120%_, P_115%_, P_110%_ and P_105%_= maximum dose of 120%, 115%, 110% and 105% of the prescribed dose, respectively.

**Table 4:** TCP data in the LQ and USC models

TCP	LQ					USC			
	P_120%_	P_115%_	P_110%_	P_105%_		P_120%_	P_115%_	P_110%_	P_105%_
1-year	100 (100-100)	100 (100-100)	100 (100-100)	99.9^a^ (99.9-100)		100 (100-100)	100 (100-100)	100 (100-100)	99.9 (99.9-99.9)
2-year	99.9 (99.9-100)	99.6 (99.6-99.7)	99.2 (98.5-99.3)	97.5^b^ (97.2-97.9)		99.9 (99.9-100)	99.6 (99.6-99.7)	99.2 (98.5-99.3)	97.5 (97.2-98.0)
3-year	99.2^c^ (98.8-99.5)	97.5^c^ (97.3-98.0)	95.3^c^ (92.7-95.9)	89.5^c^ (88.5-90.8)		99.0 (98.3-99.3)	96.6 (96.4-97.3)	93.9 (90.7-94.7)	86.8 (85.6-88.4)

Data were presented as median (range). ^a^ statistically significant with *p* value < 0.05 compared with the USC model for 1-year TCP prediction. ^b^ statistically significant with *p* value < 0.05 compared with the USC model for 2-year TCP prediction. ^c^ statistically significant with *p* value < 0.05 compared with the USC model for 3-year TCP prediction. TCP=tumor control probability. LQ=linear quadratic model. USC=universal survival curve model. P_120%_, P_115%_, P_110%_ and P_105%_= maximum dose of 120%, 115%, 110% and 105% of the prescribed dose, respectively.

## Abbreviations

SBRT: stereotactic body radiotherapy
NSCLC: non-small cell lung cancer
LQ: linear quadratic
TCP: tumor control probability
USC: universal survival curve
RTOG: Radiation Therapy Oncology Group
mLQL: modified linear quadratic and linear
BED: biologically effective dose
